# The ‘hashtag’ K-wires configuration for the management of severe comminuted patellar fracture: the combination of tension band technique and cerclage wiring

**DOI:** 10.1308/rcsann.2024.0044

**Published:** 2024-07-31

**Authors:** NE Koukoulias, AV Vasiliadis, S Savvidou, T Dimitriadis

**Affiliations:** St. Luke’s Hospital, Thessaloniki, Greece

## Background

Patellar comminuted fracture represents a challenging procedure with several proposed fixation techniques, including tension banding, cerclage wiring, low-profile plates and headless screws.^1–3^

## Technique

**Figure 1:**
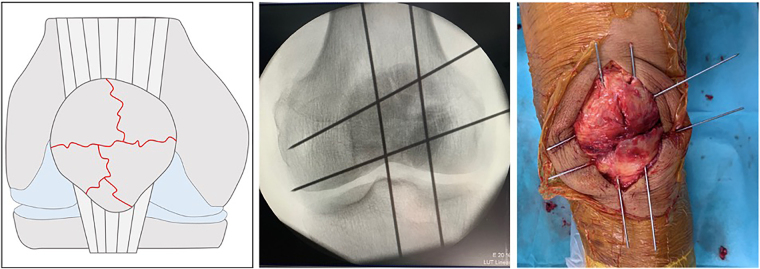
Severe comminuted patellar fracture. Intraoperative fluoroscopy image of the patient showing the ‘hashtag’ K-wire configuration.

**Figure 2:**
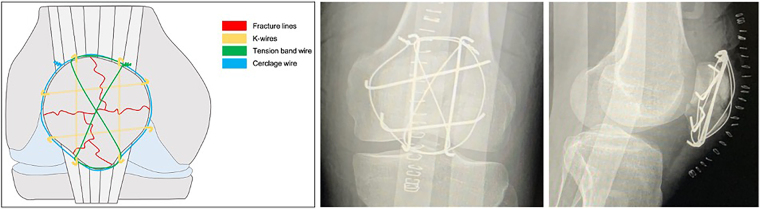
The tension band technique combined with circumferential cerclage wiring. Postoperative radiographic images of the patient showing final fixation of the severe comminuted patellar fracture.

K-wires (1.8mm) were used in a ‘hashtag’ configuration to achieve a stable fixation in comminuted patellar fractures (Figure 1). Using the outside-in technique, the first K-wire was drilled in an axial direction to reduce the two fragments of the medial side. The second K-wire was then drilled parallel to the first to reduce the two fragments of the lateral side. The third and fourth K-wires were drilled in a horizontal direction (Figure 1). It is important to verify the reduction by palpation of the joint surface. In addition, intra-operative radiographic evaluation was checked to confirm accurate reduction. A 1.2mm stainless steel wire is placed in a figure-of-eight fashion in the two axial oriented K-wires, as closed as possible to the bone and the K-wire tips. A second 1.2mm wire was then placed around the circumference of the patella body behind the horizontally oriented K-wire tips (Figure 2). At this point, the two wires were alternately tightened with the knee in extension, while the reduction was checked by palpation the articular patellar surface. Finally, the wire ends were twisted and trimmed into deeper layers to avoid any irritation.

## Discussion

This technique is based on the Arbeitsgemeinschaft für Osteosynthesefragen (AO) principles for multi-fragmentary fractures, reducing the number of fragments. Also, the combination of both techniques converts tensile force into compression force at the opposite cortex and achieves an anatomic reduction.
